# The Overlay, a New Solution for Volume Variations in the Residual Limb for Individuals with a Transtibial Amputation

**DOI:** 10.3390/s24144744

**Published:** 2024-07-22

**Authors:** Pierre Badaire, Maxime T. Robert, Katia Turcot

**Affiliations:** 1 Centre Interdisciplinaire de Recherche en Réadaptation et Intégration Sociale (Cirris)—525, Boul. Wilfrid-Hamel, Aile H Local 1300, Québec, QC G1M 2S8, Canada; pierre.badaire@protonmail.com (P.B.); maxime.robert@fmed.ulaval.ca (M.T.R.); 2 Département de Kinésiologie, Pavillon de l’Éducation Physique et des Sports, Université Laval, (PEPS) 2300, rue de la Terrasse, Local 2144, Québec, QC G1V 0A6, Canada; 3 École des Sciences de la Réadaptation, Université Laval, Pavillon Ferdinand-Vandry, 1050, Avenue de la Médecine, Local 4770, Québec, QC G1V 0A6, Canada

**Keywords:** amputation, prosthesis, volumetric variations, overlay technology

## Abstract

Background: The company Ethnocare has developed the Overlay, a new pneumatic solution for managing volumetric variations (VVs) of the residual limb (RL) in transtibial amputees (TTAs), which improves socket fitting. However, the impact of the Overlay during functional tasks and on the comfort and pain felt in the RL is unknown. Methods: 8 TTAs participated in two evaluations, separated by two weeks. We measured compensatory strategies (CS) using spatio-temporal parameters and three-dimensional lower limb kinematics and kinetics during gait and sit-to-stand (STS) tasks. During each visit, the participant carried out our protocol while wearing the Overlay and prosthetic folds (PFs), the most common solution to VV. Between each task, comfort and pain felt were assessed using visual analog scales. Results: While walking, the cadence with the Overlay was 105 steps/min, while it was 101 steps/min with PFs (*p* = 0.021). During 35% and 55% of the STS cycle, less hip flexion was observed while wearing the Overlay compared to PFs (*p* = 0.004). We found asymmetry coefficients of 13.9% with the Overlay and 17% with PFs during the STS (*p* = 0.016) task. Pain (*p* = 0.031), comfort (*p* = 0.017), and satisfaction (*p* = 0.041) were better with the Overlay during the second visit. Conclusion: The Overlay’s impact is similar to PFs’ but provides less pain and better comfort.

## 1. Introduction

Many diseases can lead to an amputation of the lower limb. Over the past decade, Canada has experienced an increase of 13% in the number of individuals undergoing lower limb amputation [1]. It is crucial for individuals with lower limb amputation to regain mobility, with prosthesis serving as a pivotal means to achieve this goal [2]. For transtibial amputees (TTA), a prosthesis typically consists of four main components, including the following: the sockets, serving as the interface between the prosthesis and the residual limb (RL); the pylon, connecting the foot to the upper portion of the prosthesis; and the prosthetic foot [3]. While these components enable TTA to engage in daily activities, the lack of musculoskeletal structures necessitates adjustments in their movement patterns.

The prosthetic gait, as observed by De Marchis et al. (2022), was marked by compensatory strategies (CSs) observed using kinematics and kinetics gait outcomes [4]. Prosthetic gait was characterized by increased step width, step length, duration of the double support phase, pelvic obliquity and trunk range of motion [4]. The primary kinetic difference from a healthy gait lies in the greater peak of vertical ground reaction forces (GRF) [4]. In a systematic review conducted by Miramand et al. (2022), it was shown that during a sit-to-stand (STS) test, TTAs rely more on their intact limb to stand up [5]. This impacted the kinematics and kinetics in the sagittal plane, with both more flexion and more vertical ground forces in the intact limb [5]. These CSs can be further exacerbated in cases of improperly aligned sockets or pylons [6,7], potentially resulting in skin, muscle and bone complications [8]. Adjustment of the socket is therefore essential for prosthesis users. However, some people experience volumetric variation (VV) of their RL during the day.

Those VVs originate from a pressure gradient caused by the socket and create a greater volume exit than entry in the interstitial spaces, leading to a decrease in RL volume [9]. Factors such as activity level, the type of socket, alimentation, and menstrual cycle can also affect the volume changing rate [10]. Standing still and walking are both linked to a volume loss. Standing generates a greater volume loss, which is mainly associated with more weight-bearing on the amputated leg [9,11]. On the other hand, while sitting, the RL tends to gain volume, because the constraints are reduced in the socket [9,12]. However, several solutions exist to make up daily adjustments for prosthesis users.

**Figure 1:**
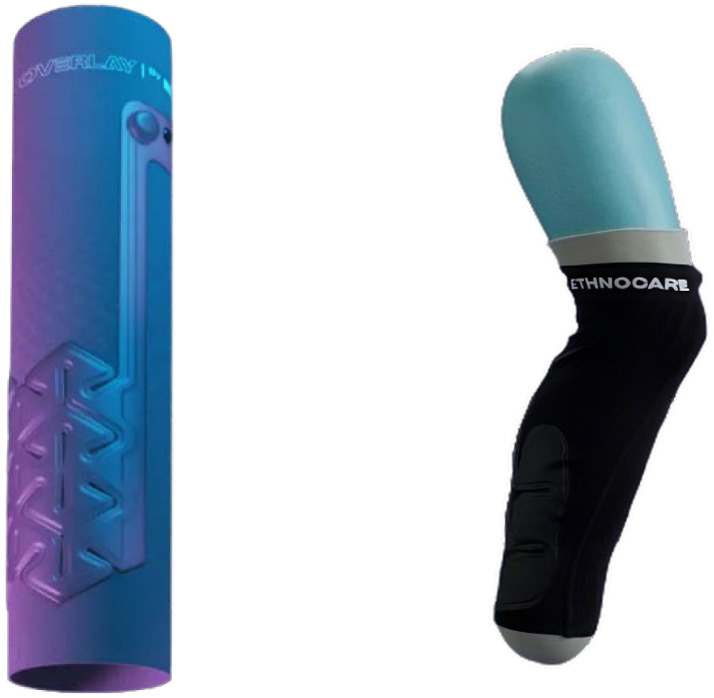
On the left, the Overlay—On the right, the Overlay worn.

One commonly employed solution to address daily volumetric fluctuations and their impacts on both kinematic and kinetic parameters is the use of prosthetic folds (PFs). PFs are sock-like devices that can be added or removed between the RL and socket to maintain proper socket fitting. It is the most common solution used to manage with VV [10]. Even though these devices are simple to use, it requires the individual to remove their prosthesis to adjust the number of folds [10]. This can be cumbersome and inconvenient in certain situations. Some people will avoid adjusting their prosthesis for convenience’s sake, and this can reduce their participation in activities [13]. Prosthetic companies have developed other solutions to cope with VV (adjustable sockets, inflatable sockets, and others) [13]. Those sockets can reduce the pressure on the residual limb, while providing similar CSs as PFs [14]. These devices are not commonly used in clinical settings, notably because they can be deemed as expensive and time-consuming to make [13]. Other promising alternatives include the use of a pneumatic bladder inside the socket to adapt the volume more easily and more precisely [15,16]. Ethnocare, a company based in Montreal (Canada) developed an innovative solution (i.e., the Overlay) using a pneumatic device (Figure 1). This device is an expandable sheath equipped with pneumatic cells, that one wears between the socket and the RL. Cells are placed on the residual calf and their number varies depending on the size of the Overlay. The air volume can be easily modified without removing the prosthesis. It is not placed inside the socket, but as the interface between the prosthetic liner and the socket. This way, it is usable with multiple kinds of socket. However, though the Overlay has been on the market since late 2022, its influence on both CSs and comfort compared to traditional PFs are still unknown.

Therefore, this study aims to measure the impact of using the Overlay compared to PFs on (1) the CS while performing functional tasks (i.e., walking, and sit-to-stand tasks), and (2) the comfort and the pain experienced during those tasks. Our hypothesis is that the Overlay will provide more comfort during the evaluated tasks and will reduce the need for CSs linked to the prosthesis. Furthermore, we would like to evaluate if the Overlay could replace PFs as a solution to cope with VV.

## 2. Materials and Methods

### 2.1. Participants

A total of 8 participants with transtibial amputation who met our inclusion and exclusion criteria were recruited. Each participant visited a prosthetist at the Institut de Réadaptation en Déficience Physique de Québec (IRDPQ) to ensure their admissibility to wear the Overlay and to receive a demonstration on how to use it. Participants’ characteristics are described in Table 1. All participants had a level of activity of at least K-3 (e.g.: being able to walk at different cadences without external assistance) [17]. One participant who was already using the Overlay was subsequently instructed to wear the PFs for two weeks prior to their initial visit. The study was approved by the Ethics Committee of CIUSSS de la Capitale-Nationale (protocol number #2022-2524) and written informed consent was obtained from all participants. The inclusion and exclusion criteria are listed in Appendix A.

### 2.2. Protocol

Each participant did two evaluations separated by an interval of two weeks. This interval was used as familiarization period for the Overlay and its duration was selected referring to previous studies [14,18]. Between the two visits, participants used the Overlay, and reported their daily activities, comfort, and pain in a daily log. A calibration trial was recorded before participants underwent the tasks of interest. During each evaluation they performed 3 tasks: sit-to-stand, gait at comfortable speed, and a 6 min walking test (6MWT) [19] under two conditions: while wearing the Overlay and while wearing PFs. The passing order of the conditions was randomized for each participant to avoid the effects of fatigue and were inverted for the second evaluation. The STS and gait were assessed at self-selected speed. Except for the 6MWT, each task was repeated to have a total of 5 proper trials recorded. A proper trial corresponded to an entire series of steps on a force plate for the gait evaluation, and for participants to stand up in a single motion with the foot always in contact of the force plates for the STS. The resting heart rate (HR) was measured prior to the evaluation session. To minimize fatigue, participants rested until their HR matched their resting HR between each task. The comfort and pain felt after each task was assessed using a visual analog scale (VAS: 0–10) by asking the participants “On a scale of 0 to 10, how would you assess the level of comfort of your prosthesis, 0 being not comfort at all and 10 being the best comfort ever” and “On a scale of 0 to 10, how would you assess the level of pain in your residual limb, 0 being not pain and 10 being the worst pain ever”.

Each participant was given the option to make adjustments between tasks. Whether using the Overlay or PFs, they could make themselves comfortable by adding or removing layers of PF or by inflating or deflating the Overlay. All participants received the same instructions before each task, which are presented in the Appendix B.

### 2.3. Materials

To capture the lower limb and trunk 3D kinematics, the participants were equipped with 44 reflective markers on both lower limbs and the pelvis, in addition to the clusters attached to each segment (i.e., shanks, thighs, trunk, sacrum, feet) following a 6 degrees-of-freedom marker set according to the International Society of Biomechanic’s standards for the lower limbs [20] and the Plug-in-gait model for the upper body (Plug-in Gait Reference Guide., 2021). The trajectories of those markers were recorded using 10 Vicon cameras (Vantage V5, Oxford, UK—100 Hz). Gait spatio-temporal parameters during the whole 6MWT were recorded with one Inertial Measurement Unit (IMU) on each foot (Physilog 4, Mindmaze, Lausanne, Suisse—200 Hz for the accelerometer and the gyroscope, 40 Hz for the magnetometer and 25 Hz for the barometer), and with the motion capture system during the gait at a comfortable speed. The GRF, joint power and moments of forces were recorded with four force plates AMTI placed on the ground, and force plates on a custom instrumented chair, on the base and on the armrest (OR6, Watertown, MA, USA—1000 Hz). The HR was monitored using a Garmin wristwatch (Garmin Furerunner 45, Garmin, Olathe, KS, USA) during the entirety of the evaluation.

A shortened version of the Prosthesis Evaluation Questionnaire (PEQ) [14] was also filled out by participants at the beginning of each evaluation. This version of the PEQ is reduced to 12 questions relating to socket fit, comfort and activity to assess their 14 previous days with their current prosthesis and VV solution. This questionnaire assesses prosthesis satisfaction, comfort and stability during daily activities, fitting of the prosthesis, the feel of the weight of the prosthesis, and the ability to put on and take off the prosthesis, evaluated on a scale of 0 to 5 (ranging from ‘0 = poor’ to ‘5 = excellent’). The numerical scales for the pain and comfort were scales ranging from 0 to 10 (respectively ranging from ‘0 = no pain’ to ‘10 = unbearable pain’ and from ‘0 = absolute discomfort’ to ‘10 = best comfort ever’).

### 2.4. Data Analysis

Kinematics and kinetics data were analyzed using The Motion Monitor (Innovative Sports Training, Chicago, IL, USA) for the comfortable gait and STS tasks. They were filtered with a 6 Hz low-pass, 4th order Butterworth filter. ISB recommendations were used to compute our biomechanical model. An XYZ Cardan–Euler sequence was used to calculate the kinematics of the ankles, knees, hips and pelvis joints (with X being the sagittal plane, Y the frontal plane and Z the transverse plane). The pelvis kinematics were calculated relative to the laboratory axes and the other lower limbs joint kinematics were calculated relative to the proximal segment [21]. Moments were normalized to the body mass of each participant and estimated using the inverse dynamics equations of Newton–Euler.

Kinematics and kinetics data of the gait were normalized from 0% to 100% of the cycle, with 0% being the initial contact of the heel and 100% the next initial contact of the same heel. The sit-to-stand was normalized from 0% to 100% using visual cue to determine the start and end of the movement, based on Turcot et al. (2012) [22]. The data were then processed with a homemade Matlab script (Matlab R2022b, MathWorks Inc., Natick, MA, USA) to calculate our variables of interest. For the kinematics of the comfortable gait task, 7 gait cycles were analyzed for each participant, and 5 trials for STS. For the kinetics, we selected 3 cycles for each task.

**Figure 2:**
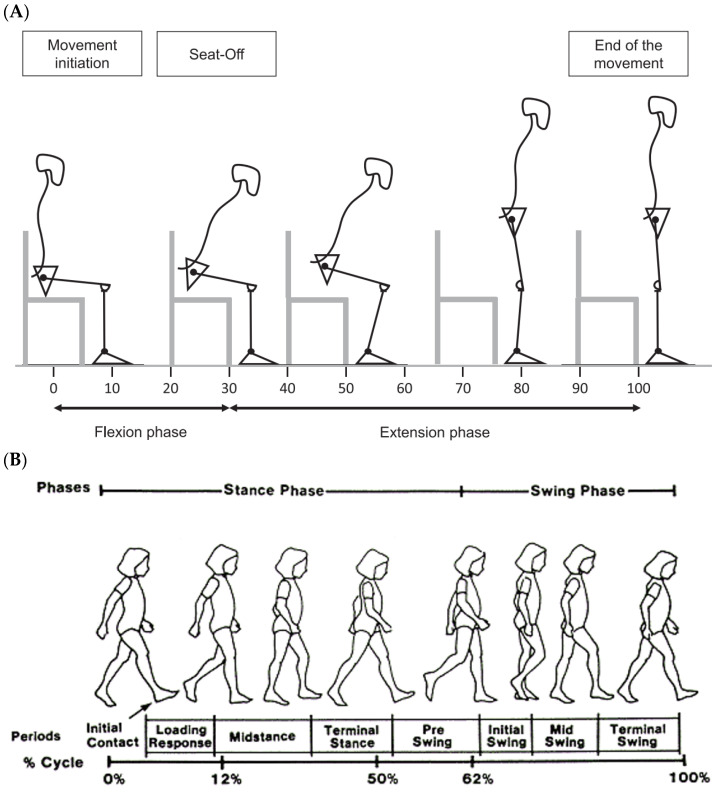
(**A**) Sit-to-stand phases (Miramand et al., 2022 [5]) (**B**) Gait cycle phases (Perry et al., 1992 [23]).

During the gait, we analyzed the range of motion of the pelvis, hip, knee and ankle of both the sound and prosthetic leg, on the sagittal plane, as well as the duration of the stance phase, and the speed, cadence, and length of step. For the sit-to-stand, we compared the range of motion on the sagittal plane, the moment of force for each joint on the sagittal plane and the GRF. The kinematics are expressed as follows: the positive values are the joint flexion, and the negative values are the joint extension. We used the phases described by Perry et al. (1992) [23] for the gait and by Miramand et al. (2022) [5] for the STS as references (Figure 2).

The data from the IMUs were analyzed with Gait Analyser software 3.1.1 (Mindmaze, Lausanne, Switzerland). Qualitative data from all questionnaires and numerical scales were compiled in an Excel file (Excel 2406, Microsoft Corporation, Redmont, WA, USA).

### 2.5. Statistical Analysis

The statistical analysis was done using the SPM1d Matlab toolbox (version M.0.4.10, www.spm1d.org (accessed on 20 May 2024)), [24] to compare the kinematics and kinetics data of each task between our two conditions. Considering the size of our sample, the normality of our data was not confirmed. Therefore, we used the nonparametric paired *t*-test, using the bootsrap and permutation method described by Pataky et al. (2015) to compare the kinematics and kinetics data provided by the SPM1d toolbox in order to evaluate the differences between the Overlay and the prosthetic PF [24]. The data from the IMUs and qualitative data were compared using a Wilcoxon test using Jamovi (The jamovi project (2023). jamovi (Version 2.3)). A significant difference was defined as *p* < 0.05.

## 3. Results

### 3.1. Comfortable Gait

During the first visit, subjects walked with an average cadence of 105 ± 7.6 steps/min with the Overlay and of 101 ± 7.16 steps/min with PFs (*p* = 0.021). This difference was not observed during the second visit (*p* = 0.093). No other spatiotemporal parameters showed significant differences between the Overlay and PFs during both visits.

**Figure 3:**
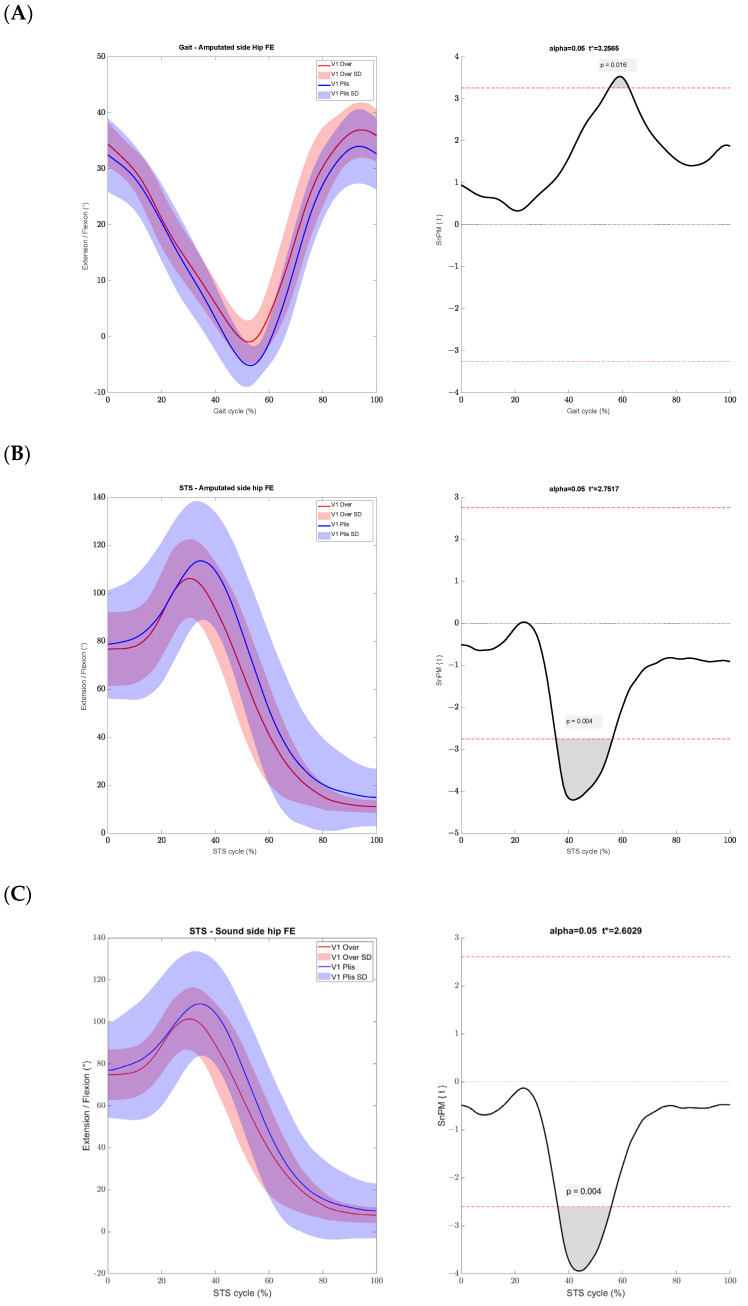
Kinematic on the sagittal plane for (**A**) the hip on the amputated side during the gait, (**B**) the hip on the amputated side during the STS, (**C**) the hip on the sound side during the STS. (**Left plot**) Kinematic under each condition. (**Right plot**) SPM Analysis, with the red dotted line as the significance threshold.

Less extension of the hip on the side of the amputation during the pre-swing phase was observed while wearing the Overlay than while wearing PFs. We found a peak extension of the hip of −2.38° ± 3.56 with the Overlay, and of −6.3° ± 3.55 with PFs (*p* = 0.016) (Figure 3). We found no significant kinematic difference between the conditions for the pelvis, knees, and ankles on both sides during the first visit. During the second visit all joints had similar ranges of motion between the two conditions. We found no significant kinetics difference between the Overlay and PFs during the two visits.

### 3.2. 6MWT Task

During the 6MWT, the distances travelled by the participants were similar during the two visits, as well as the cadence, and symmetry of the duration of the stance and swing phases. During the initial visit, it was observed that the speed was significatively higher with the Overlay during the first minute of the 6MWT when compared to PFs. We observed a speed of 1.05 m/s ± 0.23 with the Overlay and of 1.01 m/s ± 0.21 with PFs (*p* = 0.044). While this difference persisted during the second visit, it was not statistically significant (*p* = 0.166). The speeds were similar during the last minute of the test during both visits (visit 1: *p* = 0.406; visit 2: *p* = 1).

### 3.3. Sit-to-Stand Task

Significant differences for lower limb kinematics at the first visit between the Overlay and PF were observed. Both hips showed less flexion with the Overlay at the beginning of the extension phase, from 35% to 55% of the cycle. During this interval, on the amputated side the hip ranged from 104.18° to 53.26° ± 22.9 with the Overlay and from 113.5° to 67.22° ± 28.44 with PFs (*p* = 0.004). On the sound side, the hip ranged from 98.43° to 50.78° ± 23.78 with the Overlay and from 108.53° to 62.88° ± 25.53 with PFs (*p* = 0.004) (Figure 3). The kinematics of the hips, knees and ankles on both sides were similar during the second visit between the two conditions.

After normalization of the cycle, we visually observed on the data plot that the movement seemed faster with the Overlay during the two visits. Using the derivative function, we found a 2 frame difference each visit between the Overlay and the PFs even though no significant differences were found.

**Figure 4:**
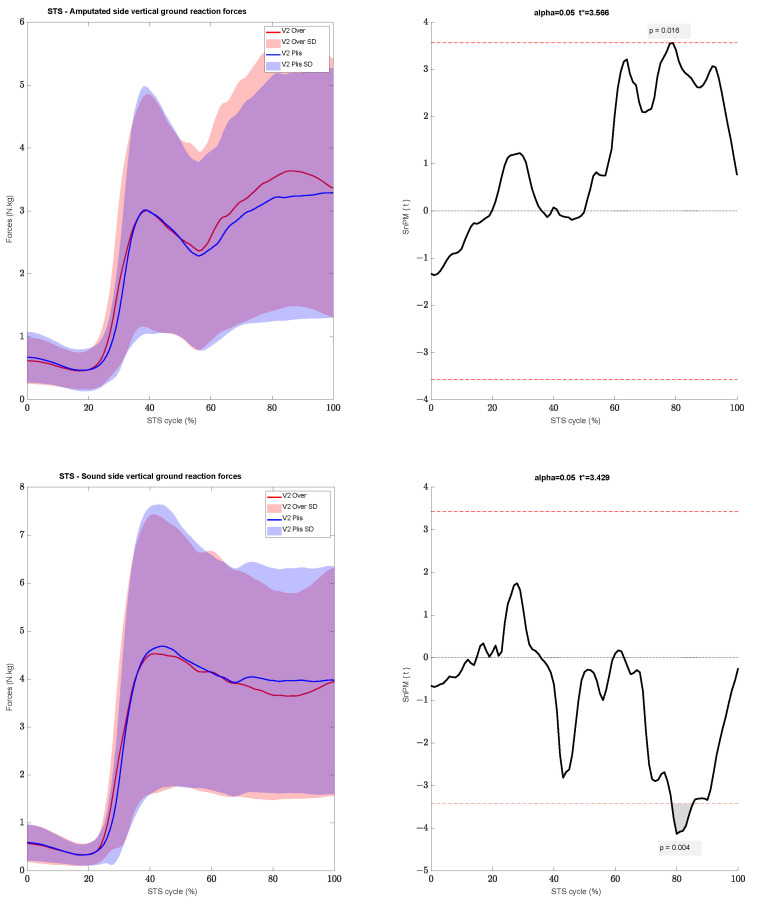
(**Left plot**) Vertical ground reaction force on both sides during the STS. (**Right plot**) SPM Analysis.

The moments of forces were similar between the two conditions and during the two visits. During the end of the STS, while stabilizing the position (i.e., between 80% and 100% of the cycle, Figure 1), we found an increase in vertical GRF with the Overlay on the prosthetic side for a small duration, at 78% of the STS cycle, with a peak of 3.43 N.kg ± 2.2 with the Overlay and 3.11 N.kg ± 2 with PF (*p* = 0.016) (Figure 4). However, the sound side showed a lesser vertical GRF at the same period but for a longer duration, from 78% to 86% of the cycle, with a peak of 3.69 N.kg ± 2.35 with the Overlay and 3.98 N.kg ± 2.51 with PFs (*p* = 0.004) (Figure 4). This was reflected in the symmetry of the forces produced between each leg even though no significant differences were found. The Overlay symmetry coefficient was 13.9% ± 11.3, while it was 17% ± 12.4 with PFs (*p* = 0.116).

### 3.4. Pain, Comfort, and Satisfaction

**Figure 5:**
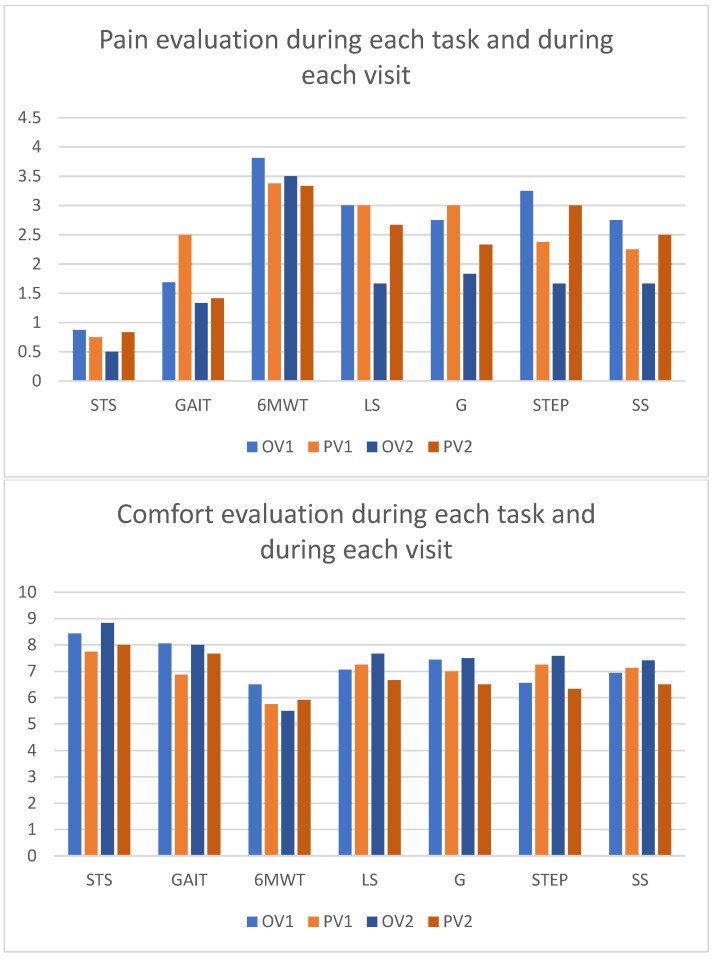
Pain and comfort evaluation for each task and during each visit. STS = sit to stand; 6MWT = six minute walking test; LS = long slope (6° incline); G = gravel; STEP = walking over a step; SS = short slope (13° incline).

During both visits and under both conditions, the most painful and the least comfortable task was the 6MWT. During the first visit, the pain felt during the 6MWT with the Overlay was evaluated at 3.81 ± 3.38 and at 3.38 ± 3.48 with PFs (*p* = 0.041), but the comfort felt between the two conditions was similar. During the second visit, the comfort during the STS was evaluated at 8.83 ± 0.75 with the Overlay and at 8 ± 0.89 with PFs (*p* = 0.042). Other tasks during each visit showed similar assessment. Pain and comfort evaluations are shown in Figure 5.

We found no differences between the Overlay and the prosthetic folds whether for the mean comfort or mean pain in the first visit. During the second visit, the mean pain felt during all tasks was 1.7 ± 0.9 with the Overlay and 2.3 ± 0.9 with PFs (*p* = 0.031) and the mean comfort felt during all tasks was 7.5 ± 1 while wearing the Overlay and 6.8 ± 0.7 with PFs (*p* = 0.017).

The modified version of the PEQ showed that the Overlay was preferred to PFs. Even though all items were evaluated similarly, the overall satisfaction of the prosthesis was at 3.81 ± 0.55 with the Overlay and at 3.16 ± 0.84 with PFs (*p* = 0.041).

The diary log indicated that the mean comfort on day 1 was rated at 7.17 ± 0.98 and improved to at 7.50 ± 1.38 by the last entry. Mean pain was initially evaluated at 3 ± 1.27 on day 1 and decreased to 2.33 ± 1.37 by the last entry. However, no significant differences were found between day 1 and the last entry of the log. The most frequently reported activity was walking, followed by walking up and down stairs. The most common issue reported was difficulties in using the Overlay, primarily noted during the first 5 days of the diary.

## 4. Discussion

The aim of this study was to compare the Overlay, a new solution for volumetric variation of the RL, to PFs. Our first results show that the Overlay does not modify the gait pattern when compared to PFs, but improves the symmetry during the STS and showed better results for comfort and pain assessment in the RL during functional tasks.

It is known that during gait, TTAs have an increased pelvis obliquity and range of motion of the trunk when compared to healthy subjects [4]. The Overlay does not impact CSs in comparison to PFs. In the present study, those joints involved showed no significant differences between the Overlay to PFs during both visits.

When it comes to temporal parameters during gait assessment, previous authors have suggested that CSs are associated with an increased duration of the stance phase on the sound limb [4]. The duration of the stance phase during comfortable gait and the 6MWT was similar between the Overlay and PFs. It seems that the Overlay does not increase these CSs either. The major difference found between the two conditions during gait assessment was the speed during the 6MWT, which was greater during the first minute of the test with the Overlay. In this regard, the Overlay allows users to walk faster during demanding tasks and get closer to a healthy gait [25]. The use of the Overlay seems not to have exacerbated gait GS and even increased the speed, a key factor in gait assessment [4,25]. However, after two weeks, the differences observed are minimal and not clinically significant. Therefore, wearing the Overlay appears to have a similar biomechanical impact on gait as wearing PFs. Based on these observations, we cannot confirm our hypothesis.

During the STS, we found interesting differences. After two weeks of familiarization, it reduces the CS with a better symmetry of the vertical GRF between the legs, a symmetry close to that of a healthy subject according to previous studies [26,27]. A healthy individual has a symmetry coefficient of 10% [27], and the Overlay was the condition which was the closest to this threshold. In the present study, this can be caused by the better knee extension strength of the prosthetic leg [26], which evenly distributes the weight between the two limbs. The Overlay could provide a more precise fitting between the RL and the prosthesis, which would allow a better transmission of the forces to extend the prosthetic leg. This better weight bearing distribution between the legs could reduce the risk of osteoarthritis and osteoporosis [8]. However, only a small region of the ground reaction forces was found statistically significant between the two conditions. Similarly, the STS results show significant differences between the Overlay and PFs, but these differences are not substantial enough to be considered clinically significant. In this regard, the Overlay appears to be comparable to the PFs, rejecting our hypothesis.

The activities involved in daily living remain a significant challenge for TTA. Our results highlight that all individuals tend to prefer the use of the Overlay on a daily basis due to the increased comfort and reduced pain. The 6MWT was the most demanding task for our group, and this was confirmed with the assessment of pain and comfort. Even though the results of the 6MWT were similar between the conditions, the Overlay showed better comfort and a reduction of pain management compared to PFs. In this regard, the Overlay seems to be more suitable for the needs of TTA during challenging tasks such as the 6MWT. Having a device that enhances socket fit could potentially encourage prosthetic users to wear their device for longer periods each day [28]. The daily log’s comfort and pain assessments showed no significant differences between the beginning and end of the diary, likely due to irregular logging by some participants over the two weeks. However, we observed that individuals could resume their usual activities without major issues after a few days of familiarization. The PEQ also shows that our subjects are more satisfied with their prosthetic leg, regarding the comfort, stability, and fitting of the prosthesis. In a previous study, Gailey et al. (2008) reported these factors as crucial for an individual with lower limb amputation to have a better quality of life [28]. The Overlay then seems to provide a better quality of life than PFs, which is why it is the preferred solution at the end of our protocol. This confirms our hypothesis.

One of the main observations of this study is the similarity in kinematics and kinetics between the two conditions after two weeks. Kinematic differences in gait and STS assessments were not observed during the second visit. Schmalz et al. (2014) demonstrated that a familiarization period is necessary to adapt to a new prosthetic device, recommending a familiarization period of three months for devices with a long learning phase. Our study suggests that a familiarization period is also necessary to properly use the Overlay. However, due to its simplicity, two weeks seems sufficient for users to become accustomed to its functioning.

Few limitations were noted. Our sample size is small, however, we have a number of participants similar to other studies investigating VV or socket fitting [29,30]. We investigated a new product, and in this regard, our results are exploratory. During our study, we only compared the Overlay to PFs (the most common solution) and not to other pneumatic solutions, such as an air bladder inside the socket.

## 5. Conclusions

The Overlay appears to be a viable alternative to prosthetic folds, as it has a similar biomechanical impact. It is, however, more comfortable and preferred to classical solutions for volumetric variations of prosthetic limbs. However, it requires some time to understand how to adjust properly in accordance with VV. Further studies should be conducted to investigate the effect of the Overlay on muscles’ activity during functional tasks and to assess its impact over a period longer than two weeks.

## Figures and Tables

**Table 1:** Participants’ description.

Characteristics	
Gender	3 Females–5 Males
Etiology	3 Traumatic–4 Medical
Age	56 (±10)
Height (m)	1.70 (±0.1)
Weight (kg)	84 (±16)
Time since amputation (year)	13 (±9)
Number of folds	5 (±2)

## Data Availability

The data presented in this study are available on request from the corresponding author.

## Appendix A

**Table A1:** Inclusion and Exclusion Criteria.

Inclusion	Exclusion
Be at least 18 years old	No stump injury
TTA	Underwent amputation at least 1 year ago
Activity level K2–K4 [17]	Harmony or thigh corset
Diameter of residual limb between 18–34 cm	Length of residual limb below knee at least 10 cm
At least 3 folds of variations during a day	84 (±16)

## Appendix B. Instructions

The following instructions were followed by a demonstration.

Gait instructions: Walk in a straight line at the same pace you would walk in an empty corridor.

STS instructions: Sit with your trunk upright, arms crossed on your chest and stand up in a single motion without moving your feet. Use the armrest in last resort.

6MWT instructions: During this test, you have to walk the greatest distance during 6 min. You have to walk and cannot run. You can accelerate or decelerate as you wish. You can take break by stopping during the test but sitting down ends the test. I will announce you have reached the 1st and 5th minute of the test. You have to walk in a 8 shape between the two cones.

Ecological tasks: You have to walk back and forth each situation 5 times.
